# Youth participation in sexual and reproductive health: policy, practice, and progress in Malawi

**DOI:** 10.1007/s00038-020-01357-8

**Published:** 2020-04-09

**Authors:** Jannah Wigle, Stewart Paul, Anne-Emanuelle Birn, Brenda Gladstone, Paula Braitstein

**Affiliations:** 1 Department of Social and Behavioural Health Sciences, Dalla Lana School of Public Health, 155 College Street, Toronto, M5T 3M7 Canada; 2 Parent and Child Health Initiative (PACHI), Lilongwe, Malawi; 3 School of Medicine, College of Health Sciences, Moi University, Eldoret, Kenya; 4 Academic Model Providing Access to Healthcare (AMPATH), Eldoret, Kenya; 5 Division of Epidemiology, Dalla Lana School of Public Health, 155 College Street, Toronto, M5T 3M7 Canada

**Keywords:** Youth, Adolescent, Young people, Reproductive health, Participation, Policymaking, Malawi

## Abstract

**Objectives:**

Ensuring youth participation in policymaking that affects their health and well-being is increasingly recognized as a strategy to improve young people’s reproductive health. This paper aimed to describe the policy context and analyze underlying factors that influence youth participation in sexual and reproductive health (SRH) policymaking in Malawi.

**Methods:**

This critical, focused ethnographic study is informed by postcolonial feminism and difference-centered citizenship theory, based on data collected from October 2017 to May 2018. Multiple research methods were employed: document analysis, focus group discussions, and “moderate” participant observation. Semi-structured interviews were conducted with key informants and youth, supplemented by open-ended drawing exercises with youth.

**Results:**

Progressive policies and the presence of youth in some policymaking structures indicate substantial headway in Malawi. However, underlying structural and societal factors circumscribe young people’s lived experiences of participation.

**Conclusions:**

Despite recent progress in involving young people in SRH policymaking, notable gaps remain between policy and practice. Recognizing and integrating young people in all stages of SRH policymaking is critical to catalyzing the social and political changes necessary to ensure their reproductive health and well-being.

**Electronic supplementary material:**

The online version of this article (10.1007/s00038-020-01357-8) contains supplementary material, which is available to authorized users.

## Introduction

Ensuring youth participation in reproductive health policymaking has been increasingly recognized by the global health community as a critical strategy to meet young people’s unique reproductive health needs (Chandra-Mouli et al. 2015). Despite international commitments and scholarship on the importance of young people’s right to participate, there is scant research on how in practice youth voices and priorities have shaped national sexual and reproductive health (SRH) policymaking and implementation of laws and policies (Villa-Torres and Svanemyr 2015; Patton et al. 2016).

This research explores the social and political contexts of youth participation in sexual and reproductive health (SRH) policymaking in Malawi. It is among the world’s first empirical studies to critically analyze the policy environment and underlying factors of young people’s participation in SRH policymaking. According to the 2018 Malawi Population and Housing Census, young people aged 10–35 years represent over 50% of the population (National Statistical Office 2018). This cohort currently experiences among the world’s highest rates of adolescent pregnancy: 29% of 15–19-year-old females are either pregnant or have children (National Statistical Office and Macro 2010). Both youth participation in social policymaking and SRH have been identified as priority areas by the Government of Malawi, exemplified by the introduction of multiple youth- and SRH-related policies over the past 2 decades (Republic of Malawi 2013, 2015).

Our study aims to generate an understanding of young people’s lived experiences of participation in SRH policymaking by describing and analyzing participatory mechanisms, the depth of their engagement, and underlying structural and societal determinants that shape their involvement. Key concepts are defined in Table 1; note that the terms “youth” and “young people” are used interchangeably.

**Table 1:** Key concepts and definitions (Malawi, 2017–2018)

Key concepts	Definition
Adolescent, youth and young people	Definitions of *adolescent, youth and young people* vary by country and region, yielding overlapping and conflicting categories. The 2016 Lancet Commission defines adolescents as being between 10 and 19 years of age, “youth” as those aged 15–24 years, and “young people” as being 10–24 years old (Patton et al. 2016). By contrast, the *African Youth Charter* (2006) and Malawi’s *National Youth Policy* (Government of Malawi 2013) define youth as individuals aged 15 to 35 and 10 to 35 years, respectively
Youth participation	The “active and meaningful involvement of young people in all aspects of their own, and their communities’ development, including their empowerment to contribute to decisions about their personal, family, social, economic and political development” (Patton et al. 2016, p. 38). It represents both a process and outcome to be involved in the institutions, structures and decisions that shape their lives (Checkoway 2011)
Youth-friendly health services (YFHS)	A package of “relevant, accessible, attractive, affordable, appropriate and acceptable” health services for young people, that aims to increase the use of health services in this population (Ministry of Health Republic of Malawi 2015, p. x)
Reproductive health	At the ICPD conference, reproductive health was defined as “a state of complete physical, mental and social well-being and not merely the absence of disease or infirmity, in all matters relating to the reproductive system and to its functions and processes” (United Nations 1995)

### Background: global context

Youth participation is recognized as a human right, as per Article 12 in the UN *Convention on the Rights of the Child* (CRC), which stipulates young people’s right to participate in decisions that affect them, including those regarding their health and well-being (UN General Assembly 1989). The CRC is frequently used to legitimize and promote youth participation within government and non-governmental institutions and processes (Richards-Schuster and Pritzker 2015). The *African Youth Charter’s* Article 11 outlines States’ commitments to ensuring youth participation in all aspects of society, including parliamentary decision-making bodies, and to developing and supporting mechanisms for youth participation at all levels of decision-making (e.g., local, national and continental) (African Union Commission 2006).

## Methods

### Theoretical framework

Two theoretical perspectives inform this study: postcolonial feminism; and difference-centered citizenship theory. Postcolonial feminism exposes and deconstructs influences of colonization, racialization, globalization, gender, and social relations that affect people’s lived experiences (Kirkham and Anderson 2002). “Third World” youth experience intersecting social categories and are often subjugated as the “Other” (Mohanty 2003), homogenized, and systemically silenced (Anderson 2004; Racine and Petrucka 2011). The second, “difference-centred” citizenship theory, acknowledges that young people should be “differently equal” and not considered inferior to adults (Moosa-Mitha 2005, p. 378; Lawy and Biesta 2006). This extends power to youth by recognizing their differences (e.g., age, gender, class) as assets (Wall 2011). Together these theories shaped the development of our research design and process (Anderson et al. 2010). While intersectionality also considers interacting axes of power, our approach provides a more robust theorization of agency and privilege (Kirkham and Anderson 2002; Moosa-Mitha 2005; Deepak 2012) by focusing on structural forces (Nash 2008; Anthias 2013; Martinez Dy et al. 2014), especially in low- and middle-income contexts.

### Research approach

A critical focused ethnography guides this exploration of young people’s lived experiences of participation in SRH policymaking in Malawi. *Focused* refers to fieldwork occurring over condensed periods of time (Wall 2014), with observation of specific events (Knoblauch 2005). Critical ethnography, informed by a postcolonial feminist perspective, recognizes the influence of multiple interacting social relations on people’s lives (Anderson 2004). This perspective allowed young people’s views to be elicited and expose structural and societal forces (Anderson 1989). The policy framework by Howlett and Ramesh (1995) was also used to identify stages of the policymaking process in which youth are engaged, including: (a) agenda-setting; (b) policy formulation; (c) decision-making; (d) implementation; and (e) monitoring and evaluation.

Multiple data generation methods were used, including document analysis; focus group discussions (FGDs); semi-structured interviews and open-ended drawing with youth; semi-structured interviews with national and district key informants, as well as community leaders; “moderate” participant observation (DeWalt and DeWalt 2011) of community, district and national policy meetings; and a reflexive fieldwork journal to record researcher’s experiences, reactions, and potential biases (Morrow 2005). Research was conducted between October 2017 and May 2018 in three districts (Nkhata Bay, Dowa and Zomba) across the northern, central and southern regions of Malawi. The lead author (JW) conducted interviews with key informants, with support from the second author (SP) for community leaders. Interviews and FGDs with youth were conducted by JW, SP and a youth researcher. Youth researchers (1 male and 1 female, aged 16–24 years for each district) were recruited based on their SRH experience and were trained and supported in research activities. SP, a Malawian with ethnographic research experience, interpreted, translated and transcribed interviews/FGDs conducted in Chichewa. JW, a Ph.D. candidate from the University of Toronto with research experience in sub-Saharan Africa, carried out all coding and analysis. All authors reviewed themes, contributed to refining and interpreting data, and critically reviewed the manuscript.

### Data generation

#### Document analysis

An initial list of policies was compiled from the *Malawi Youth Status* report (Government of Malawi 2016), a policy brief on gender and SRH policies in Malawi (Pendleton and Mellish 2015), and via governmental and non-governmental websites and publications. Criteria for policy selection included: relevance to SRH and youth participation, most recent version, and availability in English (online or printed). Eight national youth, reproductive health, health, and development policies were analyzed (Table 2).

**Table 2:** National policies on youth, sexual and reproductive health, health and development (Malawi, 2017–2018)

National policy	Definition of youth	Focus on youth in vision, goals or principles	Consideration of SRH needs of youth	Youth participate in policymaking process	Societal norms, traditions or power relations addressed	Youth considered as homogeneous group	Disaggregated data on SRH (by age and sex)
National Youth Policy (2013), Ministry of Youth and Sports (2013)	10–35 years	Yes	Yes	Yes	Yes	No	No
Malawi Youth Status Report (2016) (Government of Malawi 2016)	Report focused on youth aged 10–29 years	Yes	Yes	Yes	Yes	No	Yes
Youth-Friendly Health Services Strategy (2015–2020) (Ministry of Health Republic of Malawi 2015)	Defined as 10–35 years, but focus of YFHS is 10–24 years	Yes	Yes	Yes	Yes	No	Yes (by sex), limited disaggregation by age
National Sexual and Reproductive Health and Rights Strategy (2017–2022) (The Government of Malawi and UNFPA 2017)	15–24 years	Yes	Yes	–	No	Yes	No
National Policy for Adolescent Girls and Young Women (2018) (Malawi 2018)	10–24 years, strategy focuses on adolescent girls and young women	Yes	Yes	–	Yes	No	No
Malawi growth development strategy (MGDSIII) (2017–2022)	10–29 years (in MGDS II)	No, but youth development is cross-cutting	No	–	Yes	Yes	No
Malawi Health Sector Strategic Plan II (2017–2022)	No information	No	No	–	No	Yes	No
National strategic plan for HIV and AIDS (2015–2020)	10–24 years	No	Yes	–	Yes	No	No

#### Interviews and focus group discussions

Research participants were selected using purposive sampling methods to capture varying dimensions for youth (age, gender, socioeconomic status and level of participation) and key informants (diverse State and non-State roles). Inclusion and exclusion criteria are specified in “Electronic Supplementary Material, Appendix 1.” Interview guides were informed by a critical review of the literature and theoretical frameworks, to elicit information on power and structural inequities that shape experiences of youth participation in SRH policymaking. We recruited 46 youths aged 16–24 years (20 males; 26 females) for six FGDs, one with males and one with females in each district. We also recruited 30 youths aged 16–24 years (15 males; 15 females, across three regions) to participate in semi-structured interviews. Demographic characteristics of youth participants are summarized in “Electronic Supplementary Material, Appendix 2a and 2b.” Following each semi-structured youth participant interview, an open-ended drawing activity was used (Guillemin 2004). A total of 32 key informants were recruited: community leaders (*n* = 5), district and national policymakers (*n* = 10), and representatives from national and international civil society and non-governmental organizations (*n* = 10), and multilateral and bilateral donors (*n* = 7).

### Data analysis and interpretation

#### Document analysis

Selected policy documents were analyzed using an adaptation of the *WHO Gender Assessment Tool (GAT)* (World Health Organization 2011), previously applied to evaluate the “gender-responsiveness” of health and nutrition policies in Malawi (Pendleton and Mellish 2015; Mkandawire et al. 2018). Our adapted tool provides a rapid assessment of the overall level of “youth-responsiveness,” together with potential gaps, by comparing the definition, framing and prioritization of youth, SRH, and participation (“Electronic Supplementary Material, Appendix 3”).

#### Interviews and focus group discussions

Interview and FGD guides for youth and local community leaders were translated into Chichewa and an interpreter (SP) was used with those who did not speak English. English interview guides are available in “Electronic Supplementary Material, Appendix 4.” All interviews and FGDs were audio-recorded, with participants’ consent, and transcribed. Interviews conducted in Chichewa were translated into English. The analytic process was guided by a critical ethnographic framework, which reconstructs and analyzes local cultural systems and connects findings to broader systemic influences (Carspecken 1996).

We also engaged in latent thematic analysis to identify and synthesize themes across the data by deductively considering underlying theoretically relevant concepts, as well as inductively generating themes through fieldwork and empirical evidence (Braun and Clarke 2006). This involved familiarizing ourselves with the data and generating an initial code list based on concepts from theoretical frameworks (postcolonial feminism, difference-centred citizenship theory and stages of policymaking), and in vivo codes from detailed reading of data, including keywords, processes or events that synthesized or defined data (Coffey and Atkinson 1996). For example, “agency” represents a code generated from theory, and “speaking out” is an “in vivo code” derived from data using participants’ words. All interviews/FGDs were coded using this list; codes were simultaneously (re)defined in an iterative manner. We documented code definitions, examples of data within each code, and changes to our understandings to provide an analytic audit trail. This process identified potential relationships between codes and concepts and provided empirical support for theme construction. Related codes were grouped into broader categories, drawing from our theoretical frameworks, and supported the generation of overarching themes and theories. Summaries and memos were written to ensure transparency of coding and tracked our analytic process. Drawings were analyzed using a critical visual methodology framework, which considers participants’ descriptions and interpretations of the image (Guillemin 2004; Rose 2007). Transcripts, fieldnotes, observation notes, and memos were managed, coded, and organized using NVivo 12.

Broad criteria were employed to ensure the rigor of our approach, particularly: credibility and meaningful coherence. Credibility is demonstrated through thick description and deep interpretation of data, with results supported by concrete details and the integration of quotes. We also used multiple resources, such as theories and types of data, to explore our topic from various angles (Tracy 2010). Achieving our research objectives, connecting results to literature, and ensuring our theory, methodology, and methods were congruent also enabled production of meaningfully coherent results.

## Results

Our findings synthesize our analysis of key youth, SRH, health and development policies, as well as empirical fieldwork in Malawi. Document analysis led to a detailed baseline description of the policy landscape and analysis of interviews/FGDs generated key themes reflecting the lived experiences of youth participating in SRH policymaking, including: (1) youth participation structures; (2) youth participation in the policymaking process; (3) youth agency; (4) depth of participation; and (5) structural and societal determinants of participation.

### Policy context and landscape

Analysis of key national policies uncovers how youth and SRHR are defined and prioritized in policymaking processes in Malawi (Table 2). A lack of disaggregated data (by age/gender) is used to inform, monitor, and evaluate policymaking efforts and inadequately reflects the heterogeneity of this population. Stakeholders involved in youth SRH policymaking represent an extensive network of both State and non-State actors. However, there is a dearth of information and transparency about young people’s role in policy development. At the national level, young people represent a focus across multiple sectors (e.g., youth, gender, health, education, and HIV/AIDS), but coordination and collaboration among these stakeholders remain weak. Youth-friendly health services are funded primarily by donors, leading to inequitable distribution of services and fragmented implementation. Our analysis highlights substantial gaps across rhetoric and policies on the importance of youth and SRH, and the actual integration of youth and their perspectives in practice.

### Structures and elements of youth participation

We comprehensively identified and documented youth participatory structures in SRH policymaking (Table 3). These multi-tiered structures and the selection and representation of youth from community to national levels represent the key mechanisms involving youth in SRH policymaking processes. These forums aim to provide young people space to solicit their peers’ priorities, share youth SRH experiences with local and national decision-makers, and provide feedback to youth in communities. Participation ranges from informal opportunities to more formal or established youth structures. The majority of youth participants indicated that they were engaged in grassroots or community-level entities (e.g., local youth clubs in schools, communities, churches or donor projects). Nonetheless, only elite, highly educated and experienced youth were selected to engage in policymaking forums at national and international levels. Representatives for some structures are elected by peers (e.g., youth clubs vote at village/area/district levels).

**Table 3:** Youth participation structures across multiple structures in Malawi (2017–2018)

Level	Examples of youth participation
Community	Youth clubs (e.g., donor-led, faith-based, radio listening clubs, etc.) Youth organizations Peer education and youth community-based distribution agents^a^
Village/area	Youth representatives in village or area development committees Area youth networks
District	District youth networks Youth representatives in district policy structures (e.g., district council, district executive committee) Policy consultations at district level (through district youth office) Donor and NGO projects, meetings and structures
National	National youth parliament Membership in technical working groups across multiple ministries (e.g., youth, youth-friendly health services, HIV/AIDS) National Youth Council of Malawi Policy formulation consultations Donor and NGO projects, meetings and structures
International	Youth representatives at international meetings and forums (e.g., UN General Assembly, African Union or Commonwealth meetings)

Based on observation of policy meetings, youth interviews/focus group discussions and key informant interviews

^a^Youth community-based distribution agents are young people that distribute information and family planning commodities in their communities and among peers

### Youth participation in the policymaking process

**Fig. 1:**
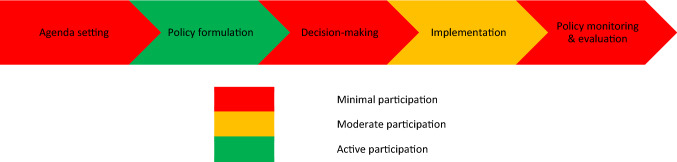
Stages of the policymaking process and youth evaluation of their level of participation (Malawi, 2017–2018)

Malawian youth emphasized that opportunities for their engagement varied across stages of the policymaking process. Most reported that their involvement was restricted to specific instances of policy formulation (e.g., writing the *National Youth Friendly Health Services Strategy 2015*–*2020*), with many considering their roles in agenda-setting, ongoing decision-making, and monitoring and evaluation to be minuscule or nonexistent (Fig. 1). Young people’s main role in the implementation of SRH policies is as peer educators and youth community-based distribution agents. These are volunteers trained in SRH and supplied select modern contraceptive methods (e.g., condoms and the contraceptive pill) to distribute among peers and community members (Republic of Malawi 2015). Young people indicated more active involvement during the policy formulation stage. However, this process was typically done by researchers or consultants, who make only marginal efforts to consult young people on their SRH priorities. Several youth interviewees reported that these consultations occurred mostly after policies were drafted, and they often did not have sufficient knowledge of English or time to review policies to contribute meaningfully. Further, many youth were not aware of key policies, such as the *National Youth Policy*, and this impeded the realization of their participatory rights.

**Fig. 2:**
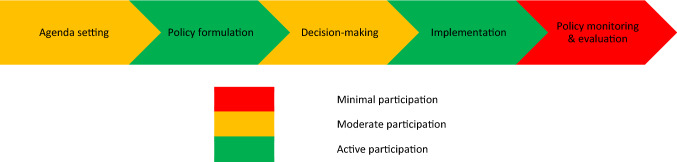
Stages of the policymaking process and policymakers’ evaluation of their level of participation (Malawi, 2017–2018)

In contrast to youth perceptions, many national and subnational policymakers claimed youth involvement to be significant throughout policymaking processes (Fig. 2). We observed adults acting as interlocutors, contributing to the exclusion or censorship of young people’s perspectives in policymaking. In addition, notwithstanding substantial rhetoric on youth SRHR and participation as a high priority, key informants emphasized that lack of funding and limits to policy implementation impeded youth participation.

“Of course, most policies are there. Malawi is one of the countries that have beautiful policies. The challenge we have in Malawi is translating the policy into action. That’s where we have a gap.” (Key Informant, National policymaker)

### Youth agency

Youth informants defined youth participation as sharing, “speaking” or “voicing out” their opinions on issues regarding youth, including SRH and overall health. They also felt that youth should be active stakeholders in the policymaking process. This was illustrated by a young person who described their role as local advocates:

“It’s not only about the youth contributing to policies but then, them also being [seen] … as drivers of change. They are supposed to take those policies and then advocate them to the fellow youth.” (Female Youth FGD participant)

Young people’s agency—as demonstrated by their motivation to improve personal and community health and well-being—increased and facilitated their participation. Young people appeared passionate about their engagement in SRH activities; disseminating SRH information through door-to-door visits, local dramas and presentations, and traveling to other communities. Young people receive financial support from their families and communities, self-fund or fundraise (e.g., growing crops) to pay for youth organization membership fees and travel costs. They indicated diverse motivations for their involvement, including that it was their duty to support community and national development, or to be a role model. Individual experiences of abuse and child marriage were also cited, as youth felt it was their responsibility to ensure that others did not face similar harms. Recognition of efforts, provision of allowances, and opportunities for experience or future employment were also key incentives. One male youth FGD respondent summarized youth agency, *“we just do most of the things on our own to help ourselves.”* Despite substantial motivation, various factors discussed ahead continue to limit opportunities for young people’s engagement in SRH policymaking.

### Depth of youth participation

**Fig. 3:**
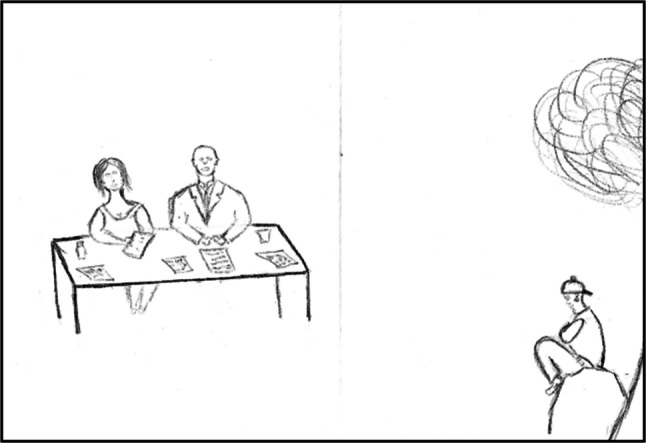
Open-ended youth drawing (Malawi, 2017–2018)

The depth and quality of young people’s participation varies widely, shaped by the type of policy activity, their level of engagement, and adults’ respect for youth. The quality of involvement ranged from symbolic or virtually no participation, to bona fide engagement. As part of the open-ended drawing exercise, one participant visually displayed their exclusion from decision-making spaces and discussions (Fig. 3) by depicting the distance of youth from the policymaking table, the power differentials between adult and youth policymakers, and the dissatisfaction of youth regarding their role.

Most youth participants considered their engagement as an important contribution to SRH policymaking, regardless of the level or form of participation. However, some young people felt disconnected with higher level policymaking and considered their grassroots roles to constitute “indirect” participation. Symbolic involvement of youth was a concern highlighted by a male youth interviewee:

“Most of the time, it’s just more like consultation. Like even myself, I’m the representative for [the] district at executive council meetings, so I’m only there to observe whatever is happening in the districts but I’m not allowed to contribute.” (Male Youth Interview Participant)

### Structural and societal determinants of participation

Many youth and key informants noted that societal expectations, such as cultural and gender norms and practices, influenced youth involvement in policymaking. Gender significantly shapes youth participation in SRH policymaking in Malawi, with young girls and women experiencing significant barriers due to experiences of harmful traditional practices, sensitivities, or taboos associated with SRH and gender inequity. Young women’s reproductive and participatory rights are interdependent, as early marriage limits their youth-related activities: It is not socially or culturally appropriate for married, young women to continue their involvement in SRH policymaking. In addition, young women expressed discomfort in discussing SRH with community decision-makers. Girls active in SRH programs or policymaking are sometimes labeled “whores” or “prostitutes” by peers and community members, which likely discourages their participation, as described by one female youth.

“People say our youth group is full of prostitutes. As such, they don’t respect our views…maybe it’s because when we are performing dramas or visiting villages, it’s when they say, ‘these are prostitutes - what they do there’.” (Female Youth Interview Participant)

Few adolescent girls or young women hold leadership positions at community and district levels. There is a disconnect between gender equity rhetorical commitments and the actual engagement of young girls and women. Some male youth participants felt that males and females had equitable opportunities, whereas others argued that females actually had an advantage for participation, jealously indicating that girls are favored in development efforts. One male youth FGD participant emphasized mockingly, *“projects are being focused on the ‘girl child, girl child, girl child.’”*

Inadequate respect for young people’s perspectives by adult decision-makers and inequitable power dynamics are serious underlying impediments to participation. Societal norms dictate that young people must respect their elders and be involved in community development (e.g., building schools/bridges, repairing roads, planting trees and cleaning hospitals), and other local activities (e.g., digging graves). Despite young people’s efforts to participate, community leaders/adults frequently do not respect or value young people’s initiative to support their peers or contribute to policymaking.

“[Adults] ignore our opinions because we are youths and they think we are too young to think in a critical way.” (Female Youth FGD Participant)

“It’s just the fear because they know that we are powerful so they are afraid that we’ll overtake [them]…So because they are afraid of losing the power…they are not even ready to empower the young person.” (Key Informant, NGO Representative and Female Youth)

## Discussion

Our research reveals that notwithstanding substantial efforts and progress in Malawi, active and meaningful engagement of youth in SRH policymaking represents an ongoing challenge. Rhetoric around supportive policies and youth participation mechanisms has not effectively catalyzed equitable opportunities, deep engagement or integration of young people across the levels and stages of policymaking. Young people’s lived experiences of participation in SRH policymaking vary substantially. At community, subnational and national levels, youth were frequently excluded or experienced symbolic or tokenistic engagement, and decision-makers solicited their perspectives superficially and only out of obligation.

Both youth and key informants felt that young people’s engagement had led to certain tangible changes in policy and community-level translation. Examples identified included recent advocacy for legislation and constitutional amendments to increase the legal age of marriage to 18 years, and the introduction of youth-friendly health services nationally. At the community-level these efforts were related to perceived increases in contraceptive use, decreased rates of child marriages, adolescent pregnancy, and school dropouts among pregnant girls. Yet even with the involvement of youth during the policymaking process, implementation of these policies has been limited; young people continue to face substantial difficulties accessing appropriate and high-quality SRH services.

In spite of these youth perceptions, health indicators have yet to improve as a result of these efforts. Indeed, the recent *Demographic and Health Survey* reported an increase in adolescent pregnancy rates (National Statistics Office [Malawi] and ICF 2017). Moreover, many improvements cited by youth were related to the adoption or upholding of already-established laws or policies, rather than addressing new priorities proposed by youth. Although many young people felt these actions showcased decision-makers’ respect for their contributions, advocacy by youth on more sensitive issues—such as the provision of in-school SRH services or legalization of safe abortion services—have thus far garnered minimal traction. This suggests the uptake of youth perspectives is restricted to matters already “approved” by adult decision-makers and that youth-led efforts to introduce new priorities face continuing obstacles.

Our research highlights a critical gap between the widespread insistence around the need to involve young people in policy and the reality of their engagement in practice. Documenting youth participatory structures, from community to international level, is a novel contribution of our work. These represent potentially effective mechanisms for including young people in policymaking processes. The presence of local and national policymakers that are champions for youth also portend a supportive driver for ensuring youth involvement in SRH policymaking, particularly at subnational levels. However, challenges related to low prioritization and insufficient financing of youth and SRH-related activities have limited the promotion of young people as key stakeholders in policymaking spaces and processes.

Postcolonial feminism and difference-centered citizenship theory framed our analysis and interpretation of young people’s lived experiences of participation in SRH policymaking. Young people’s concurrent social identities (e.g., age, sex, marital status, geographic location, etc.), interact to shape their level and depth of participation. We found that younger youth, females, people with limited formal education, and those living in rural areas have fewer opportunities and resources to engage meaningfully in SRH policymaking. Despite these hurdles, the agency of young people in Malawi remains central to our understanding of youth as not merely passive casualties of religious or cultural traditions (Deepak 2012). As evidenced in our results, young people often consider themselves “agents of change”, and their commitment and drive to contribute to youth-related activities showcases they are an important, yet underappreciated resource within communities.

Young people’s ability to be involved in SRH policymaking is also shaped by broader societal structures, including gender norms and (lack of) respect for young people. Adolescent girls and young women are expected to occupy primarily “private spaces,” such as the home; as a result their public advocacy for youth-related issues is considered inappropriate. Research from other African contexts, for instance, Kenya, Ghana and Senegal (Arnot et al. 2009, 2012; Arnot and Swartz 2012; Crossouard and Dunne 2015), underscores that gender norms frame young people’s political participation and understanding of their citizenship. Deeper consideration of how gender shapes participation amid circumscribed power and respect for girls in a largely patriarchal society and female-focused development discourses is needed.

Unequal power relations between adults and youth curb their opportunities to participate and hamper the inclusion and respect of their voices in policymaking at all levels. Various cultural values and norms reinforce the dominance of elders in families and communities and reduce young people’s willingness and effectiveness. Some youth indicated their primary role at community level was limited to unpaid manual labor. Research on youth participation in radio listening clubs, where young people engage in debates on local and political issues found unequal power relations with adults was a significant constraint on their public and political participation (Mchakulu 2007). This “age-patriarchy” represents a substantial barrier to youth involvement and reflects age-based power differentials and adult control over young people (James and Prout 1997, pp. 58–59).

### Study limitations

This research presents a snapshot of progress of engaging youth in SRH policymaking from October 2017 to May 2018. Subsequent policy initiatives or the impact of the national election in May 2019 are not considered here. Secondly, interpretation and translation transformed our data from spoken words to written texts. During this knowledge production process, transcripts serve only as representations of events (Tilley 2003), and interpretation of local realities and identities may be inadequate (Temple and Edwards 2002).

We aimed to capture a range of perspectives across young people’s social identity, including sex, age, geographic location, education and level of participation. However, few persons with disabilities, LGBTQ youth or youth under age 18 years are represented in our study. This may have limited our capture of their particular SRH needs and experiences of participation in policymaking. Lastly, this study was conceptualized and led by JW, an adult researcher from an academic institution in the “Global North”. Reflexivity or critical self-awareness of our roles as researchers is necessary as our positionality may have influenced information shared by participants due to unequal power relations. However, the lead author’s outsider position may also have enabled participants, especially youth, to share responses that may be considered culturally inappropriate in local circles.

### Conclusion

Despite aspirational calls in the international development community for incorporating young people’s participatory rights as a critical element to addressing their SRH needs (UNFPA 2014; Laski 2015), pledges to involving young people in policymaking is just a first step. A proliferation of progressive policies in Malawi has helped establish a supportive policy environment for youth involvement in SRH policymaking. Our research highlights that the introduction of mechanisms to enable young people’s participation in SRH policymaking does not guarantee that processes are effective or that youth views are respected. Bona fide youth representation in governance structures at all levels should be introduced, alongside formal strategies to avoid tokenism. Young people should be recognized as critical stakeholders in decision-making processes through voting rights in SRH policymaking, comprehensive participation, and respect for their perspectives.

Increased accessibility of policies through translation into local languages and dissemination in youth-friendly spaces (e.g., youth clubs) would improve young people’s capacity to be engaged and hold decision-makers accountable for realizing their participatory and SRH rights. In addition, ensuring the accessibility, availability and use of appropriate youth-friendly health services is needed and may be achieved through improved linkages between participatory youth structures and health services.

Adults acting in solidarity with youth, including to challenge cultural views around young people’s restricted roles and status, will help achieve the ambitious goal of young people’s active and meaningful participation in SRH policymaking. Addressing power inequities through society-wide education relating to participatory and SRH rights of youth, will also be essential. Given that youth constitute over half of the population in many African countries, including Malawi, young people (ought to) hold significant power. Their agency and involvement in all stages of policymaking are critical to catalyzing the social and political changes necessary to ensure their reproductive health and well-being.

## Electronic supplementary material

Below is the link to the electronic supplementary material.

Supplementary material 1 (DOCX 27 kb)
