# Supplementary material for: Youth participation in sexual and reproductive health: policy, practice, and progress in Malawi
Source: Int J Public Health. 2020 Apr 9;65(4):379–89. doi: 10.1007/s00038-020-01357-8 (PMC7275026; doi:10.1007/s00038-020-01357-8)
Supplement: Supplementary file 1 — Supplementary material 1 (DOCX 27 kb) [file 38_2020_1357_MOESM1_ESM.docx]

**Electronic Supplemental Material**

**International Journal of Public Health**

**Youth Participation in Sexual and Reproductive Health Policymaking in Malawi:**

**Policy, Practice and Progress**

**Appendix 1:** Inclusion/exclusion criteria for research participants (Malawi, 2017-2018)

| **Stakeholder & Activity** | **Inclusion/Exclusion Criteria** |
| --- | --- |
| **Youth Participants**  (Focus Groups)  N=6 (total number of youth was 46) | Six group discussions were conducted, with two focus group discussions (FGDs) conducted in each of the three regions, one male and one female. A total of 46 youths, aged 16-24 years participated in the FGDs.  **Inclusion**: Youth participants were current members of local youth organizations or networks, youth representatives of the village/area development committees, district executive committees, district youth technical working groups, university student unions or groups, or national youth parliament. Youth that do not currently participate in reproductive health policymaking, including out-of-school or street youth were also included. FGDs were initially proposed to recruit 4-5 males and 4-5 females for each FGD. However, due to increased interest/responses by participants to recruitment efforts (e.g., through posters, youth researchers, and various community/local non-governmental organizations (NGOs)) this was increased to 8-10 males and 8-10 females per FGD. This change required the submission of a deviation report to the University of Toronto ethics committee, as well as a submission of an amendment, which was approved. However, this was not received in sufficient time to conduct an FGD with males in Dowa district (the second group, and this was conducted with 5 individuals). Subsequent FGDs were held with 5-10 individuals.  **Exclusion**: Young people that were under younger or older than specified age range. |
| **Youth Participants**  (Semi-structured Interviews)  N=30 | 30 semi-structured interviews were conducted, with 10 interviews in each Dowa, Nkhata Bay, and Zomba districts (5 males and 5 females each).  **Inclusion**: Criteria as described for youth participants above. Sampling included 15 males and 15 females across each of the three regions (Northern, Central and Southern) Malawi. This involved recruitment of approximately 4-5 females and 4-5 males from each of the three regions (districts).  **Exclusion**: Young people that were younger or older than specified age range. |
| **Community Leaders**  (Semi-structured Interviews)  N=5 | A total of 5 semi-structured interviews were conducted with community leaders including 2 religious leaders (1 of which was also a teacher), 1 village headwoman, 1 area development committee chair, and 1 traditional chief council member.  **Inclusion**: Leaders of a local community including leadership roles in traditional authority, sub-traditional authority, village development committee or area development committee. Individual must be in role as a community leader for no less than one year. Examples: traditional chiefs, religious leader, traditional or sub-traditional authority leader, etc.  **Exclusion**: District or national level policymakers. |
| **Civil Society & NGOs**  (Semi-structured Interviews)  N=10 | A total of 10 semi-structured interviews were conducted with representatives from national, regional and interview civil society organizations (CSOs) and NGOs.  **Inclusion**: Unpaid/paid work for a local or international non-governmental organizations, civil society organizations, or international organizations that focus on youth participation in policymaking, human rights and/or youth empowerment. Examples: Plan Malawi, Centre for Youth and Civic Education, National Institute for Civic Education Trust, Young Politicians’ Union, etc.  **Exclusion**: Civil society or NGO employees that do not currently work with youth in the above fields. |
| **Donors**  (Semi-structured Interviews)  N=7 | A total of 7 semi-structured interviews were conducted with individuals from donor/funding agencies.  **Inclusion**: Unpaid/paid employees of a bilateral or multilateral funding agency working in areas of reproductive health and/or youth participation. Examples: USAID, United Nations Democracy Fund, and UNICEF Malawi.  **Exclusion**: Donor-funded civil society organizations and NGOs. |
| **District and national policymakers**  N=10 | A total of 10 semi-structured interviews were conducted with district and national policymakers, including 4 district policymakers and 6 national policymakers.  **Inclusion**: Unpaid/paid decision-maker in district or national government structures including District Executive Committee, District Youth Officers (DYO), Youth Technical Working Groups (TWG) (district & national), Ministry of Youth Labour, Youth and Manpower, and the Reproductive Health Directorate. Individuals must have been a policymaker at district/national level within the last year, for a period of no less than 1 year.  **Exclusion**: Community leaders, representatives from international organizations or NGOs. |
| **Total Participants** | **108 individuals** |

**Appendix 2a:** Demographics of FGD Youth Participants (Malawi, 2017-2018)

| **Characteristic** | **Number of Individuals** |
| --- | --- |
| **Age** | Average Age Both Sexes: 21  Average Age Female: 20  Average Age Male: 22  Range of ages: 16-24 years |
| **Gender** | Girls: 26  Boys: 20 |
| **District** | Dowa: 8 females, 5 males  Nkhata Bay: 8 females, 8 males  Zomba: 10 females, 7 males |
| **Level of Education** | Lowest level of education achieved: Form 2 (two years of post-secondary education)  Maximum level of education achieved: currently studying undergraduate degree (4th year) |
| **Marital Status** | Unmarried: 33  Married: 0  Unknown: 13 (the demographic form used in Dowa district did not ask this question) |
| **Youth with Children** | No children: 40  1 child: 5  2 children: 1 |
| **TOTAL** | **46** |

**Appendix 2b**: Demographics of youth interview participants

| **Characteristic** | **Number of Individuals** |
| --- | --- |
| **Age** | Average Age Both Sexes: 22  Average Age Female: 21  Average Age Male: 22  Range of ages: 18 years - 24 years |
| **Gender** | Girls: 15  Boys: 15 |
| **District** | Nkhata Bay: 10  Dowa: 10  Zomba: 10 |
| **Level of Education** | Lowest level of education achieved: Form 2 (2 years of post-secondary education)  Maximum level of education achieved: currently studying undergraduate degree (3rd year) |
| **Marital Status** | Unmarried: 29  Married: 1 |
| **Youth with Children** | No children: 25  1 child: 4  2 children: 1 |
| **TOTAL** | **30** |

**Appendix 3:** World Health Organization (WHO) Gender Assessment Tool & Adapted Youth Policy Analysis Tool (Malawi, 2017-2018)

**WHO Gender Assessment Tool**

1. Do the vision, goals or principles have explicit commitment to promoting or achieving gender equality?
2. Does the policy or program include sex as a selection criterion for the target population
3. Does the policy or program clearly understand the difference between sex and gender?
4. Does the target population purposely include both women and men?
5. Have women and men participated in the following stages (design, implementation, monitoring and evaluation)?
6. Have steps to ensure equal participation of women and men?
7. Do both male and female team members have an equal role in decision-making
8. Does the policy or program consider life conditions and opportunities of women and men?
9. Does the policy or program consider and include women’s practical and strategic needs?
10. Have the methods or tools been piloted with both sexes?
11. Does the policy or program consider family or household dynamics, including different effects and opportunities for individual members, such as the allocation of resources or decision-making power within the household?
12. Does the policy or program include a range of stakeholders with gender expertise as partners, such as government-affiliated bodies, national or international NGOs or community organizations?
13. Does the policy or program collect and report evidence by sex?
14. Is the evidence generated by or informing the policy or program based on gender analysis?
15. Does the policy/program consider different health needs for women and men?
16. Does the policy/program include quantitative and qualitative indicators to monitor women’s and men’s participation?
17. Does the policy or program consider gender-based division of labour (paid/unpaid/productive/reproductive)?
18. Does the policy/program address gender norms, roles and relations?**
19. Does the policy/program exclude (intentionally not) one sex but assume the conclusions apply to both sexes?
20. Does the policy/program exclude one sex in areas that are traditionally thought of as relevant only for the other sex, such as maternal health or occupational health?
21. Does the policy/program treat women and men as homogenous groups when there are foreseeable, different outcomes for subgroups, such as low-income versus high-income women or employed versus unemployed men?
22. Do materials or publications portray men/women based on gender-based stereotypes?
23. Does the language exclude or privilege one sex?

*Source:* (World Health Organization 2011)

**Adapted Youth Policy Analysis Tool**

1. What is the definition of youth in the policy/document?
2. Are youth a focus in the policy/document’s vision, goals or principles?
3. Is there a consideration of the SRH needs of youth in the policy/document?
4. Have youth participated in the policymaking process (e.g., design, implementation, monitoring and evaluation)?
5. Does the policy or program consider/address societal norms, traditions or power relations between youth and adults/decision-makers?
6. Are youth considered as a homogenous group?
7. Does the policy/program include quantitative and qualitative indicators on SRH disaggregated by age and/or sex?

**Appendix 4:** Interview guides for focus group discussions with youth, semi-structured interviews with youth and key informant interviews (Malawi, 2017-2018)

**Focus Group Discussion Guide for Youth (English)**

***General Introduction***

Thank you very much for participating in this focus group discussion. My name is ______________, I am a research assistant and interpreter and will be conducting this focus group with the principal investigator of this study, Jannah Wigle, a PhD student from the University of Toronto, Canada. This study is investigating young people’s participation in reproductive health policymaking in Malawi. You have been asked to participate as you are a young person aged 16-24 years with diverse experiences of being involved in community, district, or national policymaking efforts. Our focus group discussion today will be only *[young women/men]*, and another session will be held with *[young women/men]*. To understand the diverse dimensions and context of young people’s experiences of participation in reproductive health policymaking interviews I am also conducting focus groups in two other districts and regions, as well as conducting interviews with members of civil society, community leaders, district and national policymakers, and other young people. I will also be observing community, district and national policy meetings.

Your name and any specific community organization names will not be used in the study, please feel free to respond openly and honestly. We ask that you keep each other’s identities, participation and responses private. I cannot be sure that other participants will not share information about you with others.

This focus group will last approximately 60-90 minutes, however, you may choose to withdraw at any time or decide not to share information in the group context. Any discussions or information shared before this time will still be used. If you withdraw from the study, you will be given an opportunity to retract any statements that you have made. With your permission, I will audio record our discussion today. Your personal information and any data generated will be stored for three years after the completion of this study. Do you have any questions before we start?

***Capacity to Consent*** *[after individuals have read the consent form & I have read the above introduction]*

Before we start, I have a few questions about the consent form that you just read.

- Please explain in your own words what you just read?
- Why is signing this form is important?
- How will the information you share be used?
- Do you understand that you can withdraw at any time, or not respond to specific questions?

***Demographic & Social Characteristics***

- Let’s start by finding out a little bit more about each other by going around the room, and say your name and something about yourself.
- How old are you?
- What grade/level of education are you in or have you most recently finished?
- Why are you interested in participating in this focus group discussion?

***Local Understanding of Key Concepts: Word Association***

1. What words do you associate with the term “youth participation”?

*Probe: What about youth citizenship? Governance? Human rights?*

1. Can you describe what these concepts mean to you? How do you think these concepts are related?

***Youth Participation in Reproductive Health Policymaking***

1. In what ways are you involved in reproductive health policymaking?

*Probe: What kind of activities have you participated in? (youth clubs, youth networks, youth organizations, youth centres, etc.?) Where do you participate specifically? How often do you participate in these activities? Tell me about what activities this involves? How long have you been a part of ___________ (e.g., youth organization, national youth parliament)? What motivates this involvement?*

1. What motivates this involvement in reproductive health policymaking?

*Probe: How did you start to be involved? What motivates you to continue to participate? Why don’t you participate?*

***Structural and Societal Factors***

1. What are some of the challenges or obstacles that you have faced to participating in reproductive health policymaking?

*Probes: Is there anything in particular that you find difficult or frustrating about participating? Is logistics including time off work/timing of policy meetings/cost/availability of transportation an issue? Previously discouraging results from participation – e.g. other efforts were ignored, or did not achieve significant change? Concerns over stigma of being involved or advocating for reproductive health issues? Do you feel supported by your family?*

1. In your opinion, how do factors like gender or education level influence young people’s opportunities to participate in Malawi? Please be specific.

*Probe: What about marital status? Ethnicity? Connections or who you know? Where you live? Do young women feel comfortable speaking about reproductive health issues in front of decision-makers and community members?*

1. In what ways do policymakers value your contributions and those of other young people in policymaking, and what aspects do you think are valued less?

*Probe: Tell me about the responses you receive to your suggestions or feedback? Does this vary by community/district/national level?*

1. What changes do you think are needed to the current policies and practices to address your health and reproductive health needs?

*Probe: Are there youth-friendly health services available? More opportunities to participate?*

1. How do you understand your role as a citizen in Malawi?

*Probe: Do you think policymakers and community leaders view young people as citizens?*

***Lessons Forward***

1. What approaches do you think might facilitate active and meaningful youth participation in policymaking in Malawi?

*Probes: Do you think training on policymaking, participation and advocacy is needed? Equal opportunities for young people? Support and recognition from policymakers? Open invitations for all young people to actively participate? Is there anything that young people can do to improve their participation?*

***Conclusion***

1. What else would you like to share about your experiences in participating in reproductive health policymaking that we haven’t covered?
2. Do you have any questions for me about my project?

*Thank you very much for participating my research study Do you have any questions about the research and how the findings will be used?*

**Semi-Structured Interview Guide for Youth (English)**

***General Introduction (to be read to participants)***

Thank you very much for your participation in this interview and study. The purpose of this study is to explore youth participation in reproductive health policymaking in Malawi. To understand young people’s diverse experiences of participation in reproductive health policymaking I am conducting interviews with young people such as yourself, community leaders, civil society, non-governmental organizations, donors, and district and national policymakers. I am also holding six focus group discussions with youth in three districts in the Northern, Central, and Southern regions of Malawi and observing community, district and national policymaking meetings.

Your responses will remain anonymous, please feel free to respond openly and honestly with your thoughts to my questions. Information discussed will be analyzed, however your name or anything that can identify you will not be used in the study. You may choose to skip questions or end the interview at any time. If you decide to withdraw from the study you will still be reimbursed for your travel costs and receive refreshments.

At the end of the interview, I will ask you to make a drawing of your ideas about your participation in policymaking in Malawi. You may decide not to draw. This interview is expected to last between 60-90 minutes. With your permission, I will audio record our discussion today. Your personal information and any data generated will be stored for three years after the completion of this study. Do you have any questions before we start?

***Capacity to Consent [after reading consent form]***

Before we start, I have a few questions about the consent form that you just read.

- Please explain in your own words what you just read?
- Why is signing this form is important?
- How will the information you share be used?
- Do you understand that you can withdraw at any time, or not respond to specific questions?

***Demographic & Social Characteristics***

- Can you tell me a little bit about yourself?
- How old are you?
- What grade/level of education are you in or have you most recently finished?
- Why are you interested in participating in this interview?

***Lived Experiences of Participation***

1. In what ways are you involved in reproductive health policymaking?

*Probe: What kind of activities have you participated in? (youth clubs, youth networks, youth organizations, youth centres, etc.?) Where do you participate specifically? How often do you participate in these activities? Tell me about what activities this involves? How long have you been a part of ___________ (e.g., youth organization, national youth parliament)? What motivates this involvement?*

*Probes (If respondent indicates that they do not currently participate): What types of activities do you engage in? Please be specific, for example do you work? Do you have a family? Help at home?*

1. What motivates this involvement in reproductive health policymaking?

*Probe: How did you start to be involved? What motivates you to continue to participate? Why don’t you participate?*

1. Can you tell me about any experiences where you felt you (or your youth club), made a difference to reproductive health policies or services?

*Probes: When and where was this? What actions did this involve? What happened?*

***Structural and Societal Factors***

1. What are some of the facilitators and obstacles that you have faced to participating in reproductive health policymaking?

*Probes: Why was this helpful to you? How did this support you in your efforts? Is there anything in particular that you find difficult or frustrating about participating? Is logistics including time off work/timing of policy meetings/cost/availability of transportation an issue? Previously discouraging results from participation – e.g. other efforts were ignored, or did not achieve significant change? Concerns over stigma of being involved or advocating for reproductive health issues? Do you feel supported by your family?*

1. In your opinion, how do factors like gender or education level influence young people’s opportunities to participate in Malawi? Please be specific.

*Probe: What about marital status? Ethnicity? Connections or who you know? Where you live? Do young women feel comfortable speaking about reproductive health issues in front of decision-makers and community members?*

1. From your experience, what aspect of the policymaking process are young people involved in?

*Probe: Are you involved in deciding what is going to be discussed at meetings? Are you consulted on specific issues or concerns? Are you involved in setting the budget? Are you surveyed for national policies? Are you asked to help advocate and work with the community?*

1. In what ways do policymakers value your contributions and those of other young people in policymaking, and what aspects do you think are valued less?

*Probe: Tell me about the responses you receive to your suggestions or feedback? Does this vary by community/district/national level?*

1. In your opinion, what changes are needed to the current policies and practices to address your health and reproductive health needs?

*Probe: Are there youth-friendly health services available? More opportunities to participate?*

1. How do you understand your role as a citizen in Malawi?

*Probe: Do you think policymakers and community leaders view young people as citizens?*

***Lessons Forward***

1. What approaches do you think might facilitate active and meaningful youth participation in policymaking in Malawi?

*Probes: Do you think training on policymaking, participation and advocacy is needed? Equal opportunities for young people? Support and recognition from policymakers? Open invitations for all young people to actively participate? Is there anything that young people can do to improve their participation?*

1. What changes do you think are needed to the current policies and practices to address your health and reproductive health needs?

*Probe: Are there youth-friendly health services available? More opportunities to participate?*

***Open-ended drawing (approximately 10-15 minutes)***

1. We have talked a lot about the progress and challenges that you personally faced while being involved in reproductive health policymaking and discussed key topics including participation, citizenship and policymaking. Sometimes people also find it helpful to think about these experiences in pictures or images and not just words. For the last part of the interview, I would like you to create an image of what it is like to participate in reproductive health policymaking. You can draw about anything you want, for example about how you feel about participating, who you work with and where. It can be as simple or detailed as you like.

*Probe: How would you describe your drawing? Why did you choose these colours/placement/composition for your drawing?*

***Demographic & Social Characteristics (if not previously indicated during the interview)***

- Do you work or have a part-time or full-time job? If yes, doing what?
- Do you have any children? If yes, how many?
- Who you live with, and who is in your family?
- What responsibilities do you hold in your family and your community?

***Conclusion***

- What else would you like to share about your experiences in participating in reproductive health policymaking that we haven’t covered?
- Do you have any questions for me about my project?

*Thank you very much for participating my research study. Now that we have completed the interview are you still happy for the information shared today to be used for my research? Do you have any questions about the research and how the findings will be used?*

**Semi-Structured Interview Guide for Key Informants (English only)**

***General Introduction (to be read to participants)***

Thank you very much for your participation in this interview and study. This study explores youth participation in reproductive health policymaking in Malawi. To understand the diverse dimensions and context of young people’s experiences of participation in reproductive health policymaking interviews I am conducting interviews with members of civil society, community leaders, district and national policymakers, and young people. I am also holding six focus group discussions with youth in three districts in the Northern, Central, and Southern regions of Malawi and observing community, district and national policymaking meetings.

Your responses will remain anonymous, please feel free to respond openly and honestly with your thoughts and perspectives to my questions. Information discussed will be analyzed, however your name and exact job title will not be used in the study, and you may choose to skip questions or withdraw from participating at any time.

This interview is expected to last between 60-90 minutes. With your permission, I will audio record our discussion today. Your personal information and any data generated will be stored for three years after the completion of this study. Do you have any questions before we start?

***Background Information***

1. What is your current title/position and how long have you worked in this role?
2. Can you tell me about your interactions with young people that are participating in reproductive health policy making?

*Probe: What is your role/your organization’s role?*

***Context of Youth Participation***

1. In what ways are young people involved in the development and implementation of reproductive health policies in Malawi? Can you give specific examples?

*Probe: What kinds of activities do young people in Malawi do to participate? Are they currently involved in village/area development committees, district structures, and/or national bodies? What motivates this involvement?*

1. From your experience, what aspect of the policymaking process are young people involved in?

*Probe: Are they involved in deciding what is going to be discussed at meetings? Are they consulted on specific issues or concerns? Are they involved in setting the budget? Are they consulted on setting national policies? Are they asked to help advocate and work with the community?*

1. In your opinion, what kind of emphasis or priority is given to youth participation in reproductive health policymaking in Malawi?

*Probe: When did it become a priority? Why do you think this happened? Is this led nationally or do you think it is more internationally/donor-driven?*

***Structural and Societal Factors***

1. What are some of the facilitators and obstacles to involving young people in reproductive health policymaking?

*Probes: Financial/budget? International/national political will? Implementation plans? Status of youth in society? Logistics including time off school or work/timing of policy meetings/cost/availability of transportation an issue? Previously positive/discouraging results from participation – e.g., efforts were ignored, or did/did not achieve significant change? Concerns over stigma of being involved or advocating for reproductive health issues? Family support?*

1. In your opinion, how do factors like gender or education level influence young people’s opportunities to participate in Malawi? Please be specific.

*Probe: What about marital status? Ethnicity? Connections? Where young people live? Do young women feel comfortable speaking about reproductive health issues in front of decision-makers and community members?*

1. In what ways do you think young people’s contributions are valued in policymaking, and what aspects do you think are valued less?

*Probes: Do you feel that young people’s perspectives and opinions are respected? What kind of actions are taken as a result of engaging young people? What kinds of responses do you give to young people’s suggestions or feedback? Do you think this varies by community/district/national level?*

1. In your opinion, how has the engagement of young people influenced reproductive health policies and services?

*Probes: Do you have any specific examples you can share?* *Can you tell me about a time that you believe that young people made a difference to reproductive health policies/services through their involvement?* *If no change – why do you think that is the case? Does this vary by local/district/national levels?*

1. How do you understand young people’s roles as a citizen in Malawi?

*Probe: Do you think policymakers and community leaders view young people as citizens?*

***Lessons Forward***

1. What approaches do you think might facilitate active and meaningful youth participation in policymaking in Malawi?

*Probes: Budget? Guidelines? Training for policymakers on how to engage young people? Do you think training on policymaking, participation and advocacy is needed for youth? Equal opportunities for young people? Increased support and recognition from policymakers? Open invitations for all young people to actively participate?*

1. What kinds of activities do you think young people in Malawi can do to participate with the government and in policy?

*Probes: At community? District? National levels? What strengths/skills do you bring that would help?*

1. What else do you think young people in Malawi can do to improve their participation in reproductive health policymaking?

*Probes: At community? District? National levels? What strengths/skills do you bring that would help?*

1. What changes do you think are needed to the current policies and practices to address young people’s health and reproductive health needs?

*Probe: Are there youth-friendly health services available? More opportunities to participate?*

***Conclusion***

1. What else would you like to share about your experiences in participating in reproductive health policymaking that we haven’t covered?
2. Do you have any questions for me about my project?

*Thank you very much for your participation, your input is extremely valuable and will be used to help inform current processes for youth participation at local, district, national levels in Malawi and beyond. Now that we have completed the interview are you still happy for the information shared today to be used for my research? Do you have any questions about the research and how the findings will be used? Would you like to receive a copy of the study findings?*
